# Pratique de l’électroconvulsivothérapie dans un hôpital universitaire Tunisien: The practice of electroconvulsivotherapy at a University Hospital in Tunisia

**DOI:** 10.11604/pamj.2018.29.6.11887

**Published:** 2018-01-04

**Authors:** Anwar Mechri, Hana Zaafrane, Monia Hadj Khalifa, Samir Toumi, Férid Zaafrane, Lotfi Gaha

**Affiliations:** 1 Service de Psychiatrie, CHU Fattouma Bourguiba de Monastir, Monastir, Tunisie; 2 Service de d’Anesthésie Réanimation, CHU Fattouma Bourguiba de Monastir, Monastir, Tunisie

**Keywords:** Electroconvulsivothérapie, Tunisie, indications, anesthésie, Electroconvulsivotherapy, Tunisia, indications, anesthesia

## Abstract

Les objectifs de cette étude étaient de décrire les modalités pratiques de l’électroconvulsivothérapie (ECT) à l’hôpital universitaire de Monastir (Tunisie). Il s'agit d'une étude rétrospective portant sur tous les patients traités par ECT à l’hôpital universitaire de Monastir entre 2002 et 2013. Le nombre des patients était de 80 patients (60 hommes et 20 femmes, d’âge moyen de 42,1±15,7 ans), représentant 1,4% de l’ensemble des patients hospitalisés en psychiatrie durant la période d’étude. Le nombre total des séances d’ECT était de 784. Le diagnostic principal était le trouble dépressif majeur isolé ou récurrent chez 50% des patients. La majorité des patients (78,8%) avait reçu une seule cure d’ECT avec un nombre moyen de séances de 8,1 ± 4,9. Le produit anesthésique le plus utilisé était le propofol (97,4%). L’énergie délivrée était comprise entre 40 et 80 joules dans 71% des cas et elle était positivement corrélée à l’âge des patients. La durée moyenne de la crise motrice était de 22,3 ± 7,2 secondes et elle était négativement corrélée à l’âge des patients. Le taux de réponse le plus élevé a été constaté au niveau des scores de dépression (64,3%). Des effets indésirables immédiats ont été mentionnés chez 51,2% des patients. Enfin, 20% des patients poursuivaient des séances d’ECT d’entretien avec un rythme uni ou bimensuel. La pratique de l’ECT à l’hôpital universitaire de Monastir reste peu développée en termes de nombre des patients et des séances d’ECT. Des efforts doivent être déployés pour promouvoir l’utilisation de cette méthode.

## Abstract (en)

This study aimed to describe the practice of electroconvulsivotherapy (ECT) at the University Hospital of Monastir (Tunisia). We conducted a retrospective study of all patients treated by ECT at the University Hospital of Monastir between 2002 and 2013. 80 patients were enrolled in the study (60 men and 20 women, with an average age of 42.1±15.7 years), accounting for 1.4% of all patients hospitalized in Psychiatry Department during the study period. The total number of ECT sessions was 784. In 50% of patients, the primary diagnosis was a major isolated or recurring depressive disorder. The majority of patients (78.8%) had undergone only one ECT session, with an average number of sessions of 8.1 ± 4.9. The most used anesthetic product was the propofol (97.4%). In 71% of cases the energy delivered was between 40 and 80 joules and it was positively correlated with patients’ age. The average duration of the motor seizure was 22.3 ± 7.2 seconds and it was negatively correlated with patients’ age. The highest response rate was found in depression scores (64.3%). Immediate adverse effects occurred in 51.2% of patients. Finally, 20% of patients continued to undergo maintenance ECT sessions on a weekly or a biweekly basis. The practice of ECT at the University Hospital of Monastir has been little developed in terms of number of patients and ECT sessions. Efforts should be made to promote the use of this method.

## Introduction

L’électroconvulsivothérapie (ECT) est définie comme une méthode thérapeutique de certaines affections psychiatriques. Elle consiste à provoquer une crise comitiale généralisée au moyen d’un courant électrique à administration transcrânienne [1]. Depuis les années 1990, cette technique thérapeutique connaît un nouvel essor et le nombre d’ECT réalisées a augmenté dans plusieurs pays [2-4]. En effet, l’ECT possède actuellement une place intéressante dans l’arsenal thérapeutique de la psychiatrie. Entourée pour sa réalisation pratique de modalités précises, elle est séduisante par son efficacité remarquable dans de nombreuses maladies psychiatriques, et particulièrement celles qui posent un problème de résistance aux traitements pharmacologiques [5-7]. Cependant, elle garde une réputation sulfureuse due d’un côté aux représentations caricaturales véhiculées par les médias et d’un autre côté à l’utilisation à tort, sans anesthésie et sans curarisation, dans le passé et les effets secondaires indéniables qui en résultaient [8]. Ces considérations expliqueraient probablement que l’ECT reste peu pratiquée en Tunisie [9]. Selon cette étude réalisée auprès du personnel médical et paramédical d’un centre hospitalo-universitaire Tunisien [9], le manque d’information et de formation concernant l’ECT étaient les principales causes de la réticence envers cette thérapie. Par ailleurs, peu de données sont disponibles sur cette méthode thérapeutique en Tunisie, mais aussi au Maghreb ou même en Afrique [4]. Au centre hospitalo-universitaire (CHU) de Monastir, l’ECT est pratiquée depuis 2002 en collaboration entre les services de psychiatrie et d’anesthésie réanimation du même hôpital. Le but de ce travail était d’établir le bilan de la pratique de l’ECT au CHU de Monastir depuis son instauration en 2002. Nos objectifs spécifiques étaient de décrire les modalités pratiques de l´ECT au CHU de Monastir et de préciser la réponse et la tolérance thérapeutique de l’ECT.

## Méthodes

### Type et population d’étude

Il s´agit d´une étude rétrospective, descriptive et analytique, portant sur l’ensemble des patients ayant été traités par ECT au CHU de Monastir entre 2002 et 2013. Tous les patients hospitalisés au service de psychiatrie du CHU de Monastir (d’une capacité de 32 lits) et traités par ECT pendant la période de l’étude ont été colligés.

### Conditions de réalisation de l’ECT

Au CHU de Monastir, les séances d’ECT sont pratiquées dans une salle spécialement aménagée pour les petits actes chirurgicaux, comportant un matériel d’urgence et de réanimation et un sismothère de type Mecta SRI, puis Spectrum 5000 (depuis 2008) délivrant un courant bref et pulsé et assurant un enregistrement simultané du tracé électrocardiogramme (ECG) et électroencéphalogramme (EEG). L’indication de l’ECT est posée par le psychiatre traitant après information et consentement du patient ou de son tuteur légal et la réalisation d’un bilan pré-anesthésie. Habituellement, les séances se déroulaient avec une rythmicité d’un jour sur deux (3 séances par semaine) selon un organigramme préalable comportant les noms des patients, les jours prévus des séances et les médecins responsables.

Les séances d’ECT se déroulent en présence d’un anesthésiste, d’un psychiatre et d’un infirmier. La liberté des voies aériennes, la prévention de la morsure de la langue et la ventilation sont assurées par une canule Copa. L’application des électrodes se fait toujours en bitemporale. Les paramètres de surveillance comportent les paramètres de la stimulation (énergie délivrée, durée de la crise motrice et électrique à l’EEG et caractéristiques de la crise), les paramètres de l’ECG, de la saturation artérielle en oxygène et de la pression artérielle lors de la séance et les paramètres du réveil (reprise de la ventilation spontanée, stabilité des constantes hémodynamiques et effets indésirables après la séance).

### Recueil des données

Les données ont été recueillies à partir des dossiers médicaux des patients. Les caractéristiques épidémiologiques, cliniques et thérapeutiques des patients ont été analysées. Le diagnostic psychiatrique et les spécifications de l’épisode actuel ont été précisés selon la classification DSM-IV [10]. Les données concernant le déroulement des séances d’ECT ont été collectées sur une fiche préétablie. Pour évaluer l’efficacité de l’ECT, les échelles suivantes étaient remplies avant et après la cure d’ECT: l’échelle abrégée d’appréciation psychiatrique ou the Brief Psychiatric Rating Scale (BPRS), l’échelle de dépression de Hamilton ou the Hamilton Depression Rating Scale (HDRD), l’échelle d’évaluation de la manie ou the Mania Assessment Scale (MAS), l’échelle d’appréciation des symptômes positifs ou Scale for the Assessment of Positive Symptoms (SAPS) et l’échelle d’évaluation des symptômes négatifs ou Scale for the Assessment of Negative Symptoms (SANS) [11]. Les deux dernières échelles étaient utilisées seulement chez les patients ayant une schizophrénie ou un trouble schizoaffectif. Pour évaluer la tolérance de l’ECT, nous nous sommes référés aux effets mentionnés sur les fiches d’anesthésie et sur les dossiers médicaux des patients.

### Analyse des données

Les données ont été analysées au moyen du logiciel SPSS version 20.0. Dans la partie descriptive, nous avons calculé des fréquences absolues et des fréquences relatives (pourcentages) pour les variables qualitatives. Nous avons calculé les moyennes et les écarts-types et déterminé les valeurs extrêmes pour les variables quantitatives. La fréquence de l’utilisation de l’ECT a été calculée par rapport au nombre total des patients hospitalisés en psychiatrie durant la période de l’étude. Pour les scores des échelles, nous avons calculé le pourcentage de réduction des scores après ECT pour déterminer le taux de réponse. Dans la partie analytique, les comparaisons de 2 moyennes ont été effectuées au moyen du test *t* de Student, et en cas de faibles effectifs par le test non paramétrique de Mann et Whitney. Les comparaisons de plusieurs (> 2) moyennes ont été effectuées au moyen du test *F* de Snedecor d’analyse de la variance paramétrique (ANOVA à un facteur) et en cas de faibles effectifs par le test *H* de Kruskall-Wallis d’analyse de la variance non paramétrique. En cas de différence significative, les comparaisons 2 à 2 ont été faites par la méthode de Bonferroni. Les comparaisons de pourcentages ont été effectuées par le test du chi-deux de Pearson ou par le test exact bilatéral de Fisher. Les liaisons entre 2 variables quantitatives ont été étudiées par le coefficient de corrélation de Pearson et en cas de non-validité par le coefficient de corrélation des rangs de Spearman. Dans tous les tests statistiques, le seuil de signification a été fixé à 0,05.

## Résultats

### Fréquence de la pratique de l’ECT

Notre population comportait 80 patients traités par ECT pendant la période d’étude, ce qui représentait 1,4% de l’ensemble des patients hospitalisés en psychiatrie durant la même période (n=5663).

### Caractéristiques des patients

**Figure 1:**
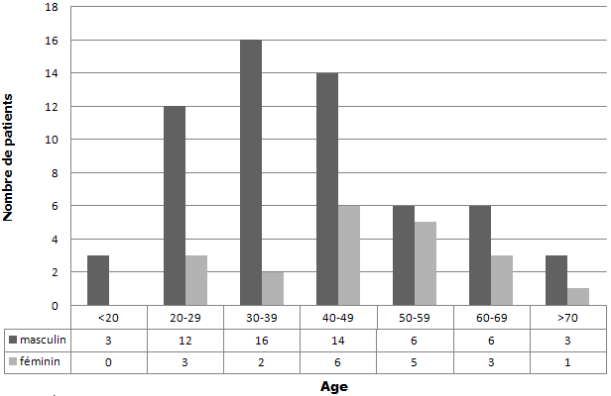
Âge à la première cure d’électroconvulsivothérapie

Lors de la première cure d’ECT, les patients avaient un âge moyen de 42,1±15,7 ans avec des extrêmes de 15 et 79 ans. Trois patients (3,7%) étaient âgés de moins de 20 ans et 13 patients (16,2%) étaient âgés de plus de 60 ans. Notre population était à prédominance masculine (75%) (Figure 1). La majorité des patients (72,5%) n’avait pas de comorbidité somatique. Pour les autres patients, les pathologies somatiques les plus fréquentes étaient l’hypothyroïdie (7,5%), le diabète (7,5%) et l’hypertension (5%). Par ailleurs, il n’y avait aucune femme enceinte parmi les patientes traitées par ECT.

L’âge moyen de début des troubles psychiatriques était de 32,8 ± 16,3 ans avec des extrêmes de 13 et 79 ans. Plus du tiers des patients (35%) avaient commis au moins une tentative de suicide (extrêmes: 1 et 7 fois). Le diagnostic psychiatrique principal était le trouble dépressif majeur (50%): épisode isolé (27,5%) ou récurrent (22,5%), suivi par la schizophrénie, le trouble schizoaffectif et le trouble schizophréniforme chez 30% des patients et le trouble bipolaire type I chez 20% des patients (Tableau 1). Pour les troubles dépressifs et bipolaires, les épisodes thymiques étaient tous sévères, avec des caractéristiques psychotiques (33,7%) ou mélancoliques (20%). La catatonie a été constatée chez 5% des patients, dans le cadre d’une schizophrénie catatonique ou d’une dépression catatonique (Tableau 1).

**Tableau 1:** Répartition selon le diagnostic psychiatrique

Diagnostic psychiatrique	Effectif	%
**Trouble dépressif majeur**	40	50
Episode isolé	22	27,5
Récurrent	18	22,5
***Spécifications de l’épisode dépressif***		
sévère	40	50
avec caractéristiques psychotiques	20	25
avec caractéristiques mélancoliques	14	17,5
avec caractéristiques catatoniques	1	1,2
**Trouble bipolaire type I**	16	20
***Nature de l'épisode actuel***		
maniaque	8	10
dépressif	4	5
mixte	4	5
***Spécifications de l’épisode actuel***		
sévère	16	20
avec caractéristiques psychotiques	7	8,7
avec caractéristiques mélancoliques	2	2,5
**Schizophrénie et autres troubles psychotiques**	24	30
Schizophrénie	12	15
sous-type indifférenciée	6	7,5
sous-type catatonique	3	3,7
sous-type paranoïde	2	2,5
sous-type désorganisée	1	1,2
Trouble schizoaffectif	10	12,5
type bipolaire	6	7,5
type dépressif	4	5
Trouble schizophréniforme	2	2,5

Au cours de l’année précédant l’ECT, le traitement psychotrope a comporté au moins un neuroleptique chez 75% des patients, un anxiolytique chez 67,5% des patients, un antidépresseur chez 56,2% des patients et un thymorégulateur chez 25% des patients. Des associations des neuroleptiques, d’anxiolytiques ou d’antidépresseurs ont été prescrites respectivement chez 55%, 38,7% et 28,7% des patients. Un traitement antérieur par ECT dans d’autres hôpitaux a été noté chez 7,5% des patients (n = 6). Cinq patients parmi eux ont rapporté qu’elle était efficace.

### Nombre de cures et de séances d’ECT

La majorité des patients (78,8%) avait reçu une seule cure d’ECT avec un nombre moyen de séances lors de cette cure de 8,1 ± 4,9 séances. Quatorze patients (17,5%) ont bénéficié d’une 2^ème^ cure d’ECT et 8 patients (10%) d’une 3^ème^ cure d’ECT. Le nombre total de cures d’ECT réalisées chez nos patients durant la période d’étude était de 102 cures et le nombre total de séances d’ECT effectuées pendant ces cures était de 784 séances. Le nombre de cures et de séances d’ECT n’étaient pas liés à l’âge ou au sexe des patients alors que le nombre moyen de cures était plus élevé chez les patients ayant un trouble bipolaire par rapport aux patients ayant un trouble schizophrénique (1,56 *vs*. 1,04; p = 0,03).

### Déroulement des séances d’ECT

La consultation pré-anesthésie a été réalisée au cours de la semaine précédant la première séance d’ECT chez 78,8% des patients. Le produit anesthésique majoritairement utilisé lors des séances d’ECT était le propofol (97,4%) associé au suxaméthonium (100%). L’étomidate était utilisé dans seulement 2,6% des séances d’ECT. L’énergie ou la charge électrique délivrée était en moyenne de 59,4 ± 26,5 joules (extrêmes: 20 et 170 joules). Elle était comprise entre 40 et 80 joules dans 71% des séances et elle était positivement corrélée à l’âge des patients (r = 0,25; p = 0,02). En revanche, il n’y avait pas de relation significative entre l’énergie délivrée et le sexe ou le diagnostic psychiatrique des patients.

La durée moyenne de la crise motrice était de 22,3 ± 7,2 secondes (extrêmes: 6 et 52 secondes) et celle de la crise électrique de 17 ± 10,5 secondes (extrêmes: 3 et 47 secondes). Il y avait une corrélation négative entre l’âge lors de la première cure et la durée de la crise motrice (r = -0,30; p = 0,006). Ainsi, plus l’âge du patient lors de la première cure d’ECT était avancé, plus la durée de la crise motrice était réduite. Par ailleurs, il n’y avait pas d’association significative entre la durée de la crise et le sexe ou le diagnostic psychiatrique des patients. De même, aucune relation significative n’a été trouvée entre la durée de la crise et l’énergie délivrée.

### Efficacité de l’ECT

Une réduction significative de tous les scores symptomatiques, à l’exception du score de la manie, a été trouvée après la première cure d’ECT (Tableau 2). Les taux de réponse les plus importants étaient constatés au niveau des scores de dépression à l’échelle HAD (64,3%) et des symptômes positifs à l’échelle SAPS (60,7%). Par ailleurs, aucune corrélation n’a été trouvée entre le taux de réponse à d’ECT et l’âge, le sexe ou le diagnostic psychiatrique des patients. De même, il n’y avait pas de relation entre le taux de réponse et l’énergie délivrée et la durée de la crise motrice ou électrique.

**Tableau 2:** Scores symptomatiques avant et après la cure d’électroconvulsivothérapie

Scores aux échelles	Moyenne	Ecart type	p
BPRS avant	43,4	15,6	**<0,001**
BPRS après	24,2	9,4	
SAPS avant	27,6	18,3	**<0,001**
SAPS après	8,8	7,4	
SANS avant	45,1	25,5	**<0,001**
SANS après	19,5	18,6	
HDRS avant	27,2	11,2	**<0,001**
HDRS après	8,2	5,1	
MAS avant	22,1	10,5	0,06
MAS après	12,0	8,9	

BPRS: l’échelle abrégée d’appréciation psychiatrique; HDRS: l’échelle de dépression de Hamilton; MAS: l’échelle d’évaluation de la manie, SAPS: l’échelle d’appréciation des symptômes positifs; SANS: l’échelle d’évaluation des symptômes négatifs

### Tolérance de l’ECT

La plupart des patients (70%) ont complété leur première cure d’ECT sans incidents graves. Aucun cas de mortalité n’a été enregistré. Pour les autres patients, la cure a été arrêtée pour des raisons techniques, médicales ou après retrait du consentement. Des effets indésirables immédiats étaient mentionnés chez 51,2% des patients, dont les plus fréquents étaient à type de bradycardie (10 cas), de dépression respiratoire (8 cas), de traumatisme dentaire (7 cas), d’encombrement bronchique (6 cas), de confusion (6 cas) et de céphalées (5 cas). La survenue de ces effets indésirables immédiats ne dépendait pas de l’âge ou du sexe des patients. En revanche, elle était associée à la durée de la crise motrice (p = 0,03) ou électrique (p = 0,02). A moyen terme, des effets cognitifs ont été rapportés à type d’amnésie rétrograde (2 cas), d’amnésie antérograde (1 cas) et de troubles de la concentration (1 cas).

### ECT de maintenance

Seize patients (20%) poursuivaient des séances d’ECT de maintenance ou d’entretien après leurs cures avec un rythme uni ou bimensuel pour chaque moitié d’entre eux. Ces patients avaient un âge moyen de 47,7 ± 15,6 ans et étaient de sexe masculin dans 12,5% de cas. Le diagnostic psychiatrique de ces patients était le trouble dépressif majeur (10 cas), le trouble bipolaire type I (3 cas), la schizophrénie (2 cas) et le trouble schizoaffectif (1 cas). La poursuite des séances d’ECT était motivée par la résistance médicamenteuse (10 cas), l’intolérance au traitement (2 cas) et la préférence du patient (1 cas).

## Discussion

Ce travail sur la pratique de l’ECT au CHU de Monastir depuis son instauration en 2002 jusqu’à 2013 a permis de dégager les caractéristiques des patients ayant bénéficié de l’ECT et de préciser les modalités techniques, l´efficacité et la tolérance de l´ECT. Toutefois, des limites méthodologiques méritent d’être relevées et prises en compte dans l’interprétation de nos résultats. Il s’agit principalement des insuffisances dans le recueil rétrospectif des données en rapport avec la maîtrise incertaine des paramètres étudiés et l’existence des examinateurs différents et de l´absence d’un suivi longitudinal des patients pour étudier l’efficacité et la tolérance de l’ECT à moyen ou à long terme.

Dans notre étude, les patients traités par ECT représentaient 1,4% de l’ensemble des patients hospitalisés en psychiatrie pendant la période d’étude. Ce taux est faible par rapport à celui rapporté par une étude similaire en Turquie menée entre 2007 et 2013 (4%) [12]. D’autres études calculant le taux de séances d’ECT par le nombre d’habitants ont montré une augmentation croissante de l’utilisation de l’ECT dans plusieurs pays [4,13]. Concernant les caractéristiques des patients, il y avait une nette prédominance masculine dans notre population. Ce résultat rejoint ceux d’autres études faites en Afrique et dans certains pays asiatiques [14,15]. Par contre, une nette prépondérance féminine a été rapportée dans les études menées en Europe, en Australie et aux Etats-Unis [13,16-19].

Dans notre série, l’âge moyen des patients lors de la première cure d’ECT était de 42,1 ans. Ce chiffre était proche de celui d’une étude Portugaise (44 ans) [18]. Toutefois, dans la plupart des études faites dans les pays occidentaux, l’ECT était le traitement préférentiel des sujets âgés de plus de 50 ans [13,16,20]. Par contre, elle était essentiellement utilisée chez l’adulte jeune (20 à 40 ans), dans certains pays africains et asiatiques [13-15]. La proportion des jeunes âgés de moins de 20 ans traités par ECT était faible dans les différentes études allant de 0,2 à 6% [13]. Ainsi, le recours à l’ECT chez les jeunes est peu fréquent. Alors que chez le sujet âgé, l’ECT s’avère être une alternative thérapeutique efficace devant les risques iatrogènes fréquents des psychotropes. Ainsi, la proportion des patients âgés de plus de 65 ans traités par ECT était dans certaines études aux alentours de 40% [13,17].

Dans notre série, 37,5% des patients présentaient une ou deux pathologies somatiques dont essentiellement le diabète, l’hypothyroïdie et l’hypertension artérielle. Une fréquente comorbidité cardiovasculaire principalement, mais aussi neurologique et endocrinienne a été rapportée chez les patients traités par ECT. Ainsi, l’ECT trouve sa meilleure indication chez les sujets ayant plusieurs comorbidités somatiques et tolérant mal la pharmacothérapie [7]. Concernant le diagnostic psychiatrique, l’ECT était utilisée principalement au cours d’un trouble dépressif majeur, isolé ou récurrent, chez la moitié de nos patients; venait par la suite l’exacerbation d’une schizophrénie ou d’un trouble schizoaffectif et la récidive thymique dans le cadre d’un trouble bipolaire. Ces pathologies correspondent globalement à celles trouvées chez les patients utilisant l’ECT dans la littérature [5-7,21]. Ainsi, le trouble dépressif majeur représente la pathologie la plus fréquente chez les patients traités par ECT dans plusieurs études [3,12,16-22]. En effet, L’ECT est efficace dans les épisodes dépressifs majeurs, que l´épisode soit isolé, récurrent ou dans le cadre d’un trouble bipolaire [3,5,21].

Dans d’autres études, la schizophrénie était le premier diagnostic psychiatrique chez les patients traités par ECT [14,15,23]. En effet, l´ECT a une efficacité remarquable dans les exacerbations schizophréniques, permettant d´obtenir un soulagement rapide [24]. Un tableau catatonique a été constaté chez 5% de nos patients, surtout dans le cadre d’une schizophrénie catatonique. L’ECT est considérée comme le traitement de choix dans cette indication trouvée chez 2 à 7% des patients selon les études [15,19,23].

Dans une cure d’ECT, le nombre total de séances se situe habituellement entre 4 et 20. Dans notre étude, le nombre moyen de séances lors de la première cure était de 8,1 ± 4,9 séances. Des résultats similaires étaient trouvés dans certaines études [3,12,18]. Toutefois, pour certains auteurs [24], le nombre de séances dépend du diagnostic et de la sévérité du trouble et il serait plus élevé dans la schizophrénie que dans les troubles de l’humeur. En cas de dépression, la rémission est attendue après une moyenne de huit séances d’ECT [21]. Dans la manie, le nombre de séances nécessaires pour obtenir une rémission est souvent inférieur à celui de la dépression [5]. Toutefois, dans notre étude, le nombre moyen de séances était comparable selon le diagnostic psychiatrique, alors que le nombre moyen de cures d’ECT était plus élevé chez les patients ayant des troubles de l’humeur. Dans notre pratique, l’anesthésie générale est systématique lors des séances d’ECT. Le propofol était le principal produit anesthésique dans notre étude. Dans la littérature, le propofol est le produit le plus utilisé, néanmoins, le thiopental et l’étomidate restent utilisés dans certains pays [2,18,20,22,23,25]. Le curare utilisé dans notre étude était le suxaméthonium, ce qui est courant dans les études [2,3,20,21].

Dans notre étude, l’énergie délivrée moyenne était de 59,4 ± 26,5 joules, soit l’équivalent de 338,8 ± 151 millicoulombs (mC). Dans la littérature, l’énergie délivrée est souvent exprimée en millicoulombs et elle variait, en moyenne, entre 244,3 et 496,6 mC selon les études [12,16,26]. Dans notre étude, une corrélation positive a été trouvée entre l’âge lors de la première cure et l’énergie délivrée. En accord avec ce résultat, d’autres études ont trouvé que l’énergie délivrée est fortement corrélé avec l’âge [20, 26]. Pour d’autres auteurs [27], l’âge, mais aussi le sexe et le nombre cumulatif des séances, sont corrélés au seuil épileptogène. La durée de la crise convulsive a été longtemps considérée comme un paramètre essentiel, conditionnant l’efficacité de l’ECT. Dans notre étude, la durée moyenne de la crise motrice était de 22,3 ± 7,2 secondes. Pour espérer avoir un effet thérapeutique, une crise convulsive doit durer au moins 15 secondes sur l’EEG et doit s’arrêter net [27,28]. Toutefois, l’efficacité n’est pas nécessairement liée à la durée de la crise, car les médicaments qui abaissent la durée de la crise n’altèrent pas toujours l’efficacité de l’ECT [29]. Par ailleurs, nous avons trouvé une corrélation négative entre l’âge lors de la première cure et la durée moyenne de la crise motrice. Ce résultat n’est pas confirmé par les données de la littérature [30, 31]. Ainsi, Pridmore et al. [32] ont conclu que l’effet de l’âge sur la durée de la crise est plutôt négligeable et que seul l’effet de l’intensité du stimulus est significatif: plus l’intensité du stimulus est élevée, plus la durée de la crise est courte. Cette relation entre la durée de la crise et la charge électrique délivrée est constatée par plusieurs auteurs [27,29,33]. Pour Sackeim et al. [27], la durée de la crise dépend de la différence entre la charge électrique délivrée et le seuil épileptique. En revanche, dans notre étude, nous n’avons pas mis en évidence de relation entre la durée de la crise et l’énergie délivrée. Ceci pourrait être en rapport avec le faible effectif de nos patients et les facteurs de confusion comme les psychotropes utilisés et les produits anesthésiques administrés.

Par ailleurs, une réduction significative de tous les scores symptomatiques a été constatée à la fin de la cure d’ECT chez nos patients, à l’exception du score de manie, probablement en rapport avec le nombre réduit des patients traités pour épisode maniaque. Aucune association significative n’a été trouvée entre le taux de réponse à d’ECT et l’âge ou le sexe des patients. En effet, l’efficacité de l’ECT ne dépend pas de l’âge des patients [34]. En accord à notre résultat, d’autres auteurs n’ont pas trouvé de corrélation entre la durée des crises et l’efficacité thérapeutique [27,33]. Dans notre étude, il n’y avait pas d’association significative entre le taux de réponse à l’ECT en fonction du diagnostic psychiatrique. Toutefois, le taux de réponse était plus élevé pour les scores de dépression (64,3%). Plusieurs études ont montré que l’ECT est efficace dans tous les types d’épisodes dépressifs majeurs avec un taux de réponse de 70 à 90% [35-37]. L’ECT serait plus efficace dans les dépressions avec caractéristiques mélancoliques, psychotiques ou catatoniques [31,37].

Dans notre série, plus de la moitié des patients (51,2%) ont présenté des effets indésirables immédiats représentés essentiellement par des complications cardiovasculaires (bradycardie) et respiratoires (dépression respiratoire, encombrement bronchique) qui constituent le principal risque immédiat associé à l’administration de l’ECT. Pour les effets secondaires psychiques, le syndrome confusionnel qui a concerné 7,5% de nos patients est une complication assez fréquente, presque constante au réveil et qui s’estompe rapidement [6]. Pour les effets cognitifs à moyen terme, ils étaient rapportés par 5% de nos patients. Dans la littérature, il s’agit du principal effet secondaire de l’ECT. Il s’agit surtout des troubles mnésiques lacunaires brefs, souvent coïncidant avec le syndrome confusionnel post-critique et plus rarement des troubles mnésiques antérogrades ou rétrogrades qui peuvent persister plusieurs mois après la cure. La fréquence et à la sévérité de ces effets cognitifs sont associées à l’âge avancé, au nombre total de séances et à la charge électrique élevée [27,38]. Toutefois, nous n’avons pas mis en évidence de corrélation entre les effets secondaires immédiats et l’énergie délivrée. Ceci serait en rapport avec l’absence d’évaluation standardisée et objective des effets secondaires et surtout des effets cognitifs de l’ECT. Par ailleurs, nous avons trouvé une relation entre les effets indésirables immédiats et la durée de la crise. Dans la littérature, cette relation est controversée avec des résultats positifs [31] et d’autres négatifs [38].

Dans notre étude, 20% des patients poursuivaient des séances d’ECT de maintenance (ECT-M) après leurs cures. Bien qu’elle ne soit pas encore bien codifiée, l’ECT-M permet de consolider le bénéfice thérapeutique obtenu et de prévenir les récidives [39]. L’ECT-M réduit la fréquence et la durée des hospitalisations et diminue le risque de rechute tout en gardant une bonne tolérance cognitive [39].

## Conclusion

Notre travail, le premier mené en Tunisie sur la pratique de l’ECT, a permis d’établir le bilan de l’utilisation de l’ECT au CHU de Monastir sur plus de dix ans. Il ressort que la pratique de l’ECT au CHU de Monastir bien qu’elle répond aux standards internationaux, reste peu développée en termes de nombre des patients et des séances d’ECT réalisées, concerne des indications classiques dominées par les troubles dépressifs chez l’adulte, avec un taux de réponse satisfaisant. Des efforts doivent être déployés pour promouvoir l’utilisation de cette méthode thérapeutique dans d’autres indications à visée à la fois curative et préventive.

### Etat des connaissances actuelles sur le sujet

- L’électroconvulsivothérapie (ECT) possède une place intéressante dans l’arsenal thérapeutique de la psychiatrie et connaît un nouvel essor dans plusieurs pays;
- Peu de données sont disponibles sur cette méthode thérapeutique en Tunisie, mais aussi au Maghreb et en Afrique.

### Contribution de notre étude à la connaissance

- Le faible taux de patients traités par ECT à l’hôpital universitaire de Monastir: 1,4% de l’ensemble des patients hospitalisés en psychiatrie pendant la période d’étude;
- La pratique de l’ECT concerne des indications classiques dominées par les troubles dépressifs chez l’adulte, avec un taux de réponse satisfaisant;
- L’ECT de maintenance ou d’entretien a concerné 20% des patients qui poursuivaient des séances à un rythme uni ou bimensuel.

## Conflits d’intérêts

Les auteurs ne déclarent aucun conflit d’intérêts.
